# Editorial: Integrative oncology treatments in rehabilitation for cancer patients: challenges and unmet needs in cancer rehabilitation

**DOI:** 10.3389/fresc.2023.1248981

**Published:** 2023-08-01

**Authors:** Byeongsang Oh, Susan Carroll, Marita Morgia, Gillian Lamoury

**Affiliations:** ^1^ Northern Sydney Cancer Centre, Royal North Shore Hospital, St Leonards, NSW, Australia; ^2^ Faculty of Medicine and Health, University of Sydney, Sydney, NSW, Australia

**Keywords:** cancer rehabilitation, integrative oncology, cancer survivors (MeSH term), quality of life, depression

**Editorial on the Research Topic** Integrative Oncology Treatments in Rehabilitation for Cancer Patients

Commensurate with advances in cancer medicine is a rapidly growing number of cancer survivors and an increased demand for cancer rehabilitation services. As cancers transition from critical diagnosis to chronic medical conditions, a continuum of rehabilitation care is required. A comprehensive cancer rehabilitation concept, caring for the whole person including their physical and psychological well-being, was proposed by Dietz and Lehmann several decades ago (1, 2). However, most of the current rehabilitation services in oncology, designed for cancer survivors, follow a traditional model, focussed on caring for physical function when this is diminishing or diminished either during or after cancer treatment, rather than a focus on caring for a person's overall well-being. Contemporary cancer survivorship models involving psychosocial and physical interventions are recommended throughout the cancer journey from the time of diagnosis to terminal stages (3). Unfortunately, this need for cancer survivors to receive more comprehensive rehabilitation has not been easily accommodated in the traditional rehabilitation model of services (4). Hence, the establishment of modern comprehensive cancer rehabilitation services to meet the needs of cancer survivors with a holistic care approach, is crucial. Furthermore, a growing body of evidence has demonstrated that cancer rehabilitation has the potential to improve survival rates for cancer survivors in addition to improving their QOL. In order to address the unmet needs of cancer survivors using conventional cancer rehabilitation services, relatively new integrative oncology therapies were initiated in the USA and spread worldwide in the last three decades. Integrative oncology seeks to provide a framework for research and the delivery of evidence-based lifestyle medicine (healthy diet and exercise) and mind-body therapies (e.g., acupuncture, massage, meditation, Yoga, Tai Chi, and Qigong) alongside standard care to prevent, manage and improve cancer-related symptoms and/or treatment-related adverse effects.

In the section on research topics for integrative oncology treatments in rehabilitation for cancer patients, four studies were accepted. Of these studies, two explored the management of cancer-related depression, one explored sarcopenia parameters in bladder cancer and the other study evaluated an integrative oncology education program for survivors of childhood cancer. Wang et al. assessed the efficacy, safety, and clinical significance of acupuncture and acupressure on depression in cancer patients and reported that acupuncture and acupressure are as effective as antidepressant medications in managing depression in cancer patients and are safe to use. They also suggested that acupuncture can contribute to cancer rehabilitation programs as an adjunctive therapy by combining pharmacological and non-pharmacological interventions. Their study findings were comparable to previous study and several clinical practice guidelines that recommend the use of acupuncture and acupressure to mitigate cancer treatment-related symptoms including pain, anxiety and depression, and improve quality of life (OQL). Battat et al. evaluated rehabilitation interventions for symptoms of depression among cancer patients in Arab and Islamic nations. This study was conducted on culturally identical Arab and Islamic populations and reported that, for this population, predominantly non-pharmacological interventions such as psychological treatment, psychosocial/spiritual support services, family education support and palliative care services were used whereas worldwide, the combination of both pharmacological and non-pharmacological interventions are most commonly used. The authors identify that the political situation and religious beliefs of cancer patients in Palestine are important factors in their experience of depression in addition to the cancer prognosis and treatment. Lam et al. presented an educational program for the safe and appropriate use of Traditional Chinese Medicine (TCM) with Chinese survivors of childhood cancer and their caregivers (*n* = 94) and evaluated the relevance of the program. Their study found that the program content was perceived as useful, and in particular the components encouraging the practice of Chinese medicine exercises and dietary advice. Although the findings warrant validation with culturally and linguistically diverse communities, they provide a framework for structuring a program to educate childhood cancer survivors and their families/caregivers on the safe and effective use of integrative oncology programs. Considering that the current integrative oncology educational programs and resources are more tailored for adult cancer survivors, this study acknowledges an important issue related to the unmet needs of childhood cancer survivors in integrative oncology. Omland et al. investigated whether sarcopenia parameters (muscle strength, muscle mass and physical performance) were associated with adherence to systemic anticancer treatment in patients with bladder cancer. Although the study was conducted with a small sample size (*n* = 14) and was prematurely terminated because of the COVID-19 pandemic, results showed a trend towards an association between reduced physical performance/muscle mass and adherence to cancer treatment. Despite terminating prematurely and finding no evidence of defined sarcopenia in the participants, this study also demonstrated the challenges of conducting clinical trials in an integrative oncology rehabilitation setting with frail cancer patients. In line with previous studies, authors on this special topic identified some of the barriers and challenges to delivering optimal cancer rehabilitation care in integrative oncology settings. A primary barrier to the optimal delivery of rehabilitation care included a lack of identification of patient problems and/or lack of appropriate referral by physicians unfamiliar with the concept of rehabilitation. A recent study also suggested that health disparities are a key barrier to accessing comprehensive cancer rehabilitation services (5).

In conclusion, cancer and its treatment impair the quality of life (QOL) of cancer patients and survivors including their physical, psychological and cognitive functioning, and affect their independent daily activities. It can also result in significant personal and family, community and government healthcare costs. Hence, there is a timely need to identify and refer patients to appropriate cancer rehabilitation services to help them to prevent, manage and recover from cancer and its treatment-related adverse effects.
